# Ultrasound‐ and Enzyme‐Assisted Protein Extracts From Sprouted *Moringa oleifera* Seeds as Functional Egg Yolk Replacers in Sustainable Plant‐Based Mayonnaise

**DOI:** 10.1155/ijfo/8396925

**Published:** 2026-06-15

**Authors:** Ruth-Ann Yaa Frimpong, Nicole Sharon Affrifah, Joris Gerald Niilante Amissah, Firibu Kwesi Saalia

**Affiliations:** ^1^ Department of Nutrition and Food Science, University of Ghana, Accra, Ghana, ug.edu.gh; ^2^ Department of Food Process Engineering, University of Ghana, Accra, Ghana, ug.edu.gh; ^3^ Department of Family and Consumer Sciences, University of Ghana, Accra, Ghana, ug.edu.gh

**Keywords:** low-acid emulsions, *Moringa oleifera* protein, plant-based mayonnaise, ultrasound- and enzyme-assisted extraction

## Abstract

This study investigated the formulation of plant‐based mayonnaise through the partial substitution (5%–50%) of egg yolk with modified extracted proteins from sprouted *Moringa oleifera* (MO) seeds. The protein from MO seeds was extracted using ultrasound‐assisted extraction (UAE), enzymatic‐assisted extraction (EAE), and their combination, ultrasound‐assisted enzymatic extraction (UAEE). Extraction method significantly influenced protein functional properties, with effects on mayonnaise texture, droplet size distribution, color, pH, and emulsion stability. Substitution improved firmness, consistency, and adhesiveness compared with the control, with a threshold at 20% replacement beyond which textural attributes declined. At this optimal level, UAEE mayonnaise matched a commercial benchmark in firmness, consistency, and adhesiveness. Formulations with 5%–10% EAE, 5% UAE, and 20% UAEE exhibited smaller *D*_4,3_ and *D*_3,2_ values, indicating finer droplet size distributions. Color analysis depicted a significant (*p* value ≤ 0.05) decline in yellowness index as substitution levels increased. The pH of the mayonnaise ranged between 5.04 and 5.47 and increased minimally yet consistently with higher substitution, thus the formulated mayonnaise analogue can be classified as a low‐acid food. These findings highlight the potential of sprouted MO seed proteins as a functional ingredient in reduced‐egg mayonnaise.

## 1. Introduction

Mayonnaise is a stable, semisolid oil‐in‐water emulsion valued for its creamy texture, rich mouthfeel, and distinctive flavor profile. However, its formulation is technologically challenging because of the very high oil fraction (typically 70%–80%), which requires strong emulsifiers to maintain droplet dispersion and prevent phase separation. Therefore, it is produced by emulsifying vegetable oil with egg yolk, acidulants such as vinegar or lemon juice, and seasonings such as mustard [1, 2]. Among these, egg yolk is an important ingredient mainly due to its phospholipids (notably lecithin) and lipoproteins including lipovitellin and livetin, which adsorb rapidly at the oil–water interface and contribute to stabilizing the emulsion [3]. Additionally, phospholipids and lipoproteins also play a central role in oxidative stability and flavor release, whereas other ingredients such as mustard and acidulants contribute to texture, spreadability, and the characteristic yellow hue. Collectively, these are critical to product quality and consumer acceptance [1, 4].

Globally, mayonnaise is among the most consumed condiments, incorporated into sandwiches, salads, and dips, and as a base for sauces across diverse cuisines [2]. The global mayonnaise market continues to expand, reflecting strong consumer demand [5]. Within this growth, rising consumer interest in plant‐based diets, allergen‐free products, and sustainability concerns has created a technological challenge for egg‐free or reduced‐egg formulations [6, 7]. Consequently, the interplay of market and consumer dynamics is driving innovation toward plant proteins capable of mimicking key functional roles of egg yolk by providing emulsifying, textural, and sensory function, while maintaining overall product quality and offering allergen‐free alternatives that meet clean‐label and dietary requirements [6, 8–10].

Several plant proteins, including soy, pea, and chickpea, have been explored as egg yolk replacers in mayonnaise to create mayonnaise analogues [8, 11–13]. These innovations focused on partially replicating the yolk′s emulsifying capacity, rheological stability, and sensory appeal, particularly at moderate substitution levels (30%–50%), with outcomes at higher levels remaining challenging and inconsistent [6]. Although these formulation innovations have achieved varying degrees of success, they predominantly focus on protein extracts from widely used seeds and cereals. In contrast, underutilized seeds, which are seldom explored in such formulations, can be a source of proteins with favorable functional properties, as demonstrated in emerging studies using sesame seed cake [14], flaxseed [15], and legume derivatives such as aquafaba [7]. In the African context, *Moringa oleifera* (MO) represents a particularly underexplored seed source with promising functional proteins for mayonnaise formulations [16].

MO seeds are considered to be underutilized seeds even though they are rich in proteins (i.e., 30%–40%), essential amino acids, and bioactive compounds with antioxidant and antimicrobial activities [17–19]. Importantly, beyond protein content, MO seed proteins exhibit functional attributes relevant to mayonnaise systems, including aqueous solubility under acidic conditions ( ?70%) [20]; surface activity and emulsifying capacity (interfacial tension reduction to ~18.6 mN/m; EAI 42.3 m^2^/g at 1% protein) [21, 22]. These properties, particularly at optimal concentrations, support their potential as partial egg yolk replacers and position MO as a promising alternative protein in mayonnaise formulations. An added advantage is that sprouting MO seeds enhances nutritional and functional potential through enzymatic activity, increasing bioactive compounds, and reducing antinutrients [23, 24]. Despite these promising attributes, the application of MO seed proteins in mayonnaise, particularly for partial egg replacement, remains limited. Evidence of feasibility was reported by Bolarin and Oke, [16] who incorporated MO seed flour into mayonnaise and found that a 1% substitution level was the highest acceptable without compromising sensory quality, and highlights the need for further exploration of MO proteins in this application.

Many studies have shown that protein extraction with green methods can significantly affect functional properties, particularly interfacial activity, droplet stabilization, solubility, and emulsifying capacity [25–27]. For instance, ultrasound‐assisted extraction (UAE) uses acoustic cavitation to disrupt cell walls, increasing protein flexibility and exposing hydrophobic groups, which enhances adsorption at the oil–water interface and improves droplet stabilization [28–30]. Enzymatic‐assisted extraction (EAE) induces targeted hydrolysis, which improves protein solubility and emulsifying capacity by modifying peptide size distribution [31]. When combined, ultrasound‐assisted enzymatic extraction (UAEE) can increase protein yield and modify molecular structure to enhance emulsifying capacity and emulsion stability, which is hypothesized to be desirable in high‐oil systems such as mayonnaise, although these outcomes have not yet been fully researched [32, 33].

Building on the previous work by Bolarin and Oke [16], this study examines whether protein extraction from sprouted MO seeds using UAE, EAE, and UAEE can enhance functional performance sufficiently to support higher levels of egg yolk substitution without compromising key quality attributes. Mayonnaise formulations containing 5%–50% egg yolk substitution were evaluated for texture, droplet size, color, pH, and stability. By linking extraction methods to end‐product performance, the study provides insight into the comparative effectiveness of UAE, EAE, and UAEE across substitution levels. These findings contribute to ingredient innovation and inform strategies for reducing egg yolk in emulsified foods such as mayonnaise.

## 2. Materials and Methods

### 2.1. Materials

Freshly harvested and dried MO seeds of the PKM‐2 (Periyankulam‐2) cultivar, selected for their high seed yield and protein content, were purchased from registered smallholder farmers in Ghana. Cultivar authenticity was verified by the Department of Crop Science, University of Ghana, using morphological descriptors to confirm varietal identity. Sodium chloride (food grade), sodium hypochlorite (NaClO) (analytical grade), sodium dodecyl sulfate (analytical grade), and sodium azide (analytical grade) were purchased from Sigma Aldrich and Merck. The mayonnaise ingredients (sunflower oil, fresh egg, sugar, mustard cream, and vinegar) were purchased locally.

### 2.2. Production of Sprouted *Moringa oleifera* Seed Flour (SMOF)

According to Frimpong et al. [23], the production of SMOF was achieved through a combination of priming and sprouting treatments.The seeds were sterilized by soaking in 200‐ppm NaClO for 10 min, a treatment shown to effectively reduce microbial load while maintaining protein content and germination rate [23]. After sterilization, seeds were thoroughly rinsed with distilled water to remove residual hypochlorite. The cleaned seeds (300 g) were steeped in water (1.5 L, 25°C) for 9 h and spread on sterile, wet jute bags. They were left to sprout at room temperature (23°C–28°C) and 98*%* ± 2*%* humidity for 4 days in a germinator (Seedburo‐164918401, United States). Sprouting was conducted in the dark to prevent light interruption. Sprouting was deemed to have occurred when the rupture of the tegument and the visible elongation of the coleoptile and coleorhiza were observed. Ungerminated seeds were not included during sampling. After germination, the seeds were dried in a vacuum oven (Genlab Oven, Model: DP/100/SS/F/DIG) at 75°C for 6 h to remove moisture and halt germination. Rootlets and shoots of the grains were separated by rubbing the germinated seeds in a 0.6‐mm sieve. The sprouted seeds were milled into fine flour with a spice mill (Kenwood, Model:715737) to pass through a 0.4‐mm sieve. The MO seed flour was stored in a sterile bag at 4°C until further analysis.

SMOF used in this study had 45 g/100 g of crude protein, 36 g/100 g of fat, 4.3 g/100 g of crude fiber, 3.4 g/100 g of total ash, and 7.2 g/100 g of carbohydrate.

### 2.3. Preparation of MO Seed Protein Extracts

#### 2.3.1. UAE

UAE parameters (ultrasound power, sonication duration, and solid–liquid ratio) were optimized in preliminary optimization studies conducted in our laboratory, and targeted maximum protein yield. This is reported in separate studies currently under peer review. Briefly, UAE of proteins from SMOF was performed using an ultrasound device (UP400S, Hielscher, Germany) with a power rating of 400 W, equipped with a titanium probe (H22D, 22 mm) and run at 24‐kHz frequency. SMOF and distilled water were first mixed at a ratio of 1:20 g/mL and sonicated for 20 min, 20% amplitude at 20°C. Afterwards, the slurry was centrifuged at 3000 rpm for 10 min to separate the oil, skim (low‐fat aqueous phase), and residue. The oil phase was removed, and the skim and residue were recovered and mixed to form a slurry. The slurry was adjusted to pH 11 with 2‐M sodium hydroxide (NaOH) solution and kept at 25°C for 60 mins. The slurry was centrifuged for 10 min at 4000 rpm to recover the supernatant. The supernatant was then adjusted to pH 6 with 2 M hydrochloric acid (HCl) to precipitate proteins and microcentrifuged at 13,000 × g for 10 min to recover the precipitated proteins. The precipitates were resuspended in distilled water, 5× the weight of protein precipitate, and stirred for 30 min while adjusting to pH 7 with 2‐M NaOH. Afterwards, the precipitates were lyophilized (−35°C, 2.5 mbar, 72 h) using a Super‐Modulyo freeze dryer from Thermo Scientific, to obtain the protein extract labelled as UAE. The UAE lyophilized protein extract had moisture of 6.23 g/100 g, crude protein of 32.4 g/100 g, 0.95 g/mL^−1^ water holding capacity (WHC), 3.67 g/mL^−1^ oil holding capacity (OHC), and 60.49‐min emulsion stability index (ESI).

#### 2.3.2. Enzyme‐Assisted Extraction (EAE)

The EAE parameters (solid–liquid ratio, enzyme concentration, and hydrolysis time) were selected based on optimization studies in our laboratory. This is reported in separate studies currently under peer review. Specifically, SMOF was first mixed with distilled water (1:20 g/mL), and the pH of the mixture was adjusted to 5 with 2‐M HCl. Just after, 2.6% (*w*/*w*) of each carbohydrase enzyme, consisting of endo‐beta‐glucanase, polygalacturonase, and cellulase, was added and incubated at 45°C for 60 min in a temperature‐controlled water bath (Thermo Scientific, GP 20 series). Subsequently, the pH was adjusted to pH 8.5 using 2‐M NaOH, and held at 60°C for the protease enzyme (endopeptidase) hydrolysis for 60 min. After hydrolysis for 60 min, it was halted at 90°C for 10 min, then rapidly cooled to room temperature. The slurry was then centrifuged at 3000 rpm for 10 min to separate the oil, cream, skim, and residue. The oil phase was removed, and skim, and residue were recovered and mixed to form a slurry. The slurry was adjusted to pH 11 with 2‐M NaOH and incubated at 25°C for 60 min, then centrifuged at 4000 rpm for 10 min to recover the supernatant. The supernatant was adjusted to pH 6 with 2 M HCl to precipitate proteins, and the mixture was microcentrifuged at 13,000 × g for 10 min to recover the precipitates. The precipitates were resuspended in distilled water at 5× the protein precipitate weight and stirred for 30 min while adjusting the pH to 7 with 2‐M NaOH. Afterwards, the precipitates were lyophilized (−35°C, 2.5 mbar, 72 h) using a Super‐Modulyo Freeze Dryer from Thermo Scientific to obtain the protein extract labelled EAE. The EAE lyophilized protein extract had moisture of 3.81 g/100 g, crude protein of 14.7 g/100 g, WHC of 17.54 g/mL^−1^, OHC of 0.93 g/mL^−1^, and ESI of 60.49 min.

#### 2.3.3. UAEE

**Figure 1:**
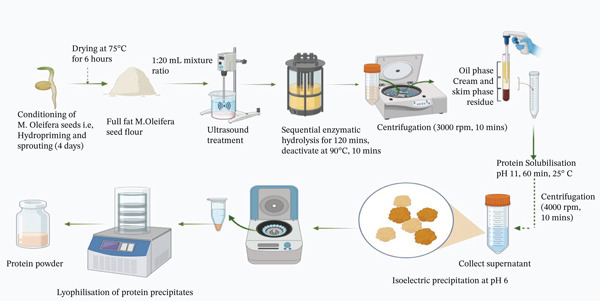
Schematic illustration of the production of UAEE proteins. Created in BioRender [23] (https://BioRender.com/3q5k4sx).

For UAEE (Figure 1), the extraction parameters (solid–liquid ratio, enzyme concentration, hydrolysis time, ultrasound power, and sonication duration,) were selected based on optimization studies in our laboratory. This is reported in separate studies currently under peer review. SMOF and distilled water were first mixed at a ratio of 1:20 g/mL and sonicated for 20 min, 20% amplitude at 20°C. The parameters for sonication were selected based on preliminary optimization studies conducted in our laboratory, targeting maximum protein yield. Afterwards, the pH of the mixture was adjusted to 5 with 2 M HCl. Just after, 2.6% (*w*/*w*) of each carbohydrase enzyme (i.e., endo‐beta‐glucanase, polygalacturonase, and cellulase) was added and incubated at 45°C for 60 min in a temperature‐controlled water bath (Thermo Scientific, GP 20 series). Subsequently, the pH and temperature were set to pH 8.5 (2‐M NaOH) at 60°C for the protease enzyme (endopeptidase). Hydrolysis was halted after 60 min at 90°C for 10 min, then rapidly cooled to room temperature. The slurry was then centrifuged at 3000 rpm for 10 min to separate the oil, cream, skim, and residue. The oil phase was removed, and cream, skim, and residue were recovered and mixed to form a slurry. The slurry was adjusted to pH 11 with 2‐M NaOH and incubated at 25°C for 60 min, then centrifuged at 4000 rpm for 10 min to recover the supernatant. The supernatant was adjusted to pH 6 using 2‐M HCl to precipitate proteins and microcentrifuged at 13,000 × g for 10 min to recover the precipitates. The precipitates were resuspended in distilled water, 5× the weight of protein precipitate, and stirred for 30 min while adjusting to pH 7. Afterwards, the precipitates were then lyophilized (−35°C, 2.5 mbar, 72 h) using a Super‐Modulyo (Thermo Scientific) to obtain the protein extract. This was labelled as UAEE. The UAEE protein extract had 1.70 g/100 g moisture, 15.00 g/100 g crude protein, WHC of 6.66 g/mL^−1^, OHC of 4.30 g/mL^−1^, and ESI of 35.99 min.

### 2.4. Mayonnaise Preparation

Mayonnaise was prepared using sunflower oil (75 g), fresh egg yolk (8 g), salt (1.5 g), sugar (1.5 g), mustard cream (1.5 g), and vinegar (5 g) according to the procedure described by Gao et al. [34] with modifications. To obtain mayonnaise products that contain MO seed proteins, the egg yolk was replaced at 5%–50% (*w*/*w*) using EAE, UAE, and UAEE lyophilized protein extracts, as shown in Table 1. Mayonnaise with 0% egg yolk substitution was used as a control. The aqueous phase containing the proteins, vinegar, sugar, and salt was homogenized for 5 min using a hand‐held mixer (HMP30, Kenwood, United Kingdom) at Speed 2. Sunflower oil was added gradually to create a concentrated emulsion. The control followed the same procedure with egg yolk as the protein used. Samples were stored at 4°C for 24 h before analysis.

**Table 1:** Mayonnaise formulations.

Substitution	Mayonnaise composition						
	Egg yolk (g)	Lyophilized Protein extract (g)	Sugar (g)	Salt (g)	Vinegar (g)	Mustard (g)	Sunflower oil (g)
0%	8	0	1.5	1.5	5	1.5	75
5%	7.6	0.4	1.5	1.5	5	1.5	75
10%	7.2	0.8	1.5	1.5	5	1.5	75
20%	6.4	1.6	1.5	1.5	5	1.5	75
50%	4	4	1.5	1.5	5	1.5	75

### 2.5. Characterization of Mayonnaise

#### 2.5.1. Droplet Size and Distribution

The size and distribution of the emulsion droplets were measured using a laser diffraction (LD) particle size analyzer (Infitek, PSA‐2L2398A), following the methods described by Mahfouzi et al. [35] with modification. The refractive indices of 1.47 and 1.33 were applied for sunflower oil and water, respectively. Samples were diluted 10 times with water before measurements [36]. The mixture was gently vortexed until complete dispersion was achieved. The volume‐weighted mean diameter (*D*_4,3_) and surface‐weighted mean diameter (*D*_3,2_) were determined to characterize oil droplet size distribution. Additionally, *D*_10_, *D*_50_, and *D*_90__,_ representing the particle diameters at the 10th, 50th, and 90th percentiles, respectively, of the cumulative particle size distribution, were determined.

The droplet size distribution breadth (span) was estimated using the span index according to Santos et al., [37] using Equation (1) below:

(1) Span=D9010−DD50

#### 2.5.2. Determination of the Texture of Model Mayonnaise

**Figure 2:**
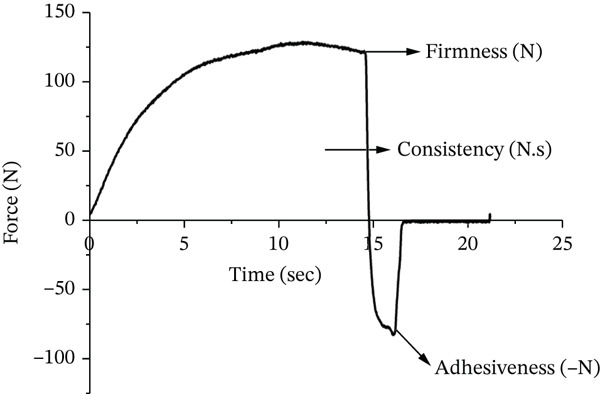
Typical textural curve derived from the back extrusion analysis of the formulated mayonnaise.

The back extrusion textural analysis procedure was conducted with a Texture Analyzer (Stable Micro System: TA‐XT2i) after storing the samples at 4°C for 24 h, according to the procedure described by Angioloni and Collar, [38]. The test was done in a back‐extrusion cell (50‐mm diameter) and a compression disc (35 mm) probe with a load cell of 5 kg. The procedure parameters used were test speeds of 1 mm s^−1^ and 25‐mm distance to measure firmness, consistency, and adhesiveness. As illustrated in Figure 2, the “peak” or maximum force was measured as firmness (*N*). The consistency (*N*.*s*) is the positive area under the curve. The negative force of the curve is the result of the weight of the sample; thus, adhesiveness is the maximum force required to lift the upper surface of the disc on return, that is, due to back extrusion, indicative of sample stickiness.

#### 2.5.3. Determination of pH of Mayonnaise

The pH values of mayonnaise were measured at 25°C using a Horiba pH meter F‐12 (Horiba Co., Japan). Three replicate readings were taken for each pH measurement of the samples.

#### 2.5.4. Determination of Color

Color measurements were performed using a Hunter Lab colorimeter according to de Araújo et al. [39]. The CIE‐Lab* color space system was employed to represent the color coordinates, with L* indicating black to white values, a* representing the red–green coordinate and b* the yellow–blue coordinate. Before measurement, the instrument was calibrated with the white and black standard plate. The yellowness index (YI) of the formulated mayonnaise was determined according to the equation reported by Ordóñez‐Santos et al. [40].

(2) Yellowness index YI=142.86×b∗L∗

where L* represents the lightness, b* represents the yellow/blue axis, and 142.86 is a constant.

#### 2.5.5. Determination of Emulsion Stability

The emulsion stability of mayonnaise samples was determined according to Jia et al. [41] with modifications. Four grams of mayonnaise samples were placed into centrifuge tubes and placed in a water bath (Thermo Scientific, GP 20 series), at 60°C for 30 min. The samples were centrifuged at 4°C (4000 r/min, 20 min), and the upper oil phase was separated. The following equation was used to calculate the emulsion stability:

(3) Emulsion stability %=F0−F1F0×100

where *F*_0_ represents the weight of the sample (g), and *F*_1_ represents the weight of the separated oil removal (g)

#### 2.5.6. Freeze–Thaw (F‐T) Characteristics of Mayonnaise

The F‐T characteristics of the mayonnaise were determined according to Huang et al. [11] with modification. One cycle of F‐T treatment involved a freezing process in a freezer at −20°C for 24 h, and a thawing process at 25°C for 2 h. After destabilization, the emulsion stability was calculated by determining the percentage of oil in the upper layer.

(4) Emulsion stability=V1V0×100%

where*V*_1_ = volume of separated oiland*V*_0_ = total sample volume.

### 2.6. Statistical Analysis

Each experiment was conducted in three independent biological batches, with each batch analyzed in triplicate technical replicates. Data were subjected to two‐way analyses of variance (ANOVA) using Minitab Version 16, and significance was established at *p* value ≤ 0.05. Within‐treatment means were separated using Tukey′s differences of means. Origin Version 19 was used to generate graphs and other data visualizations.

## 3. Results and Discussion

### 3.1. Texture of the Formulated Mayonnaise

The textural properties of the formulated mayonnaise, as measured by large deformation analysis with a back extrusion test, are presented in Table 2. These data were compared against a control sample formulated with 100% egg yolk. Additionally, the formulations were benchmarked against a commercially available full‐fat mayonnaise (commercial mayonnaise [CM]) to assess how closely the experimental textures align with an accepted product standard.

**Table 2:** Textural properties of model mayonnaise based on back extrusion analysis.

Extraction method	Substitution level (%)	Textural Properties		
		Firmness (*N*)	Consistency (*N*.*s*)	Adhesiveness (*N*)
UAE	5	90.60 ± 0.40^g^	924.17 ± 36.13^h^	−58.70 ± 0.00^g^
	10	123.40 ± 0.00^c^	1372.74 ± 8.87^e^	−79.50 ± 0.00^c^
	20	131.42 ± 0.17^b^	1522.65 ± 0.35^c^	−93.23 ± 1.05^b^
	50	46.33 ± 0.81^i^	333.43 ± 0.84^j^	−32.24 ± 0.15^d^

EAE	5	99.45 ± 0.09^f^	741.23 ± 13.11^i^	−68.17 ± 0.11^j^
	10	115.82 ± 0.00^d^	1024.51 ± 25.59^g^	−73.70 ± 0.00^d^
	20	124.06 ± 0.00^c^	1199.85 ± 12.85^f^	−79.54 ± 0.00^c^
	50	105.17 ± 0.00^e^	1438.85 ± 22.24^d^	−55.16 ± 0.00^i^

UAEE	5	87.40 ± 0.00^h^	878.14 ± 15.5^h^	−57.00 ± 0.00^h^
	10	100.00 ± 0.00^f^	914.19 ± 8.01^h^	−62.20 ± 0.00^f^
	20	167.29 ± 0.17^a^	1848.05 ± 1.91^a^	−106.40 ± 2.70^a^
	50	123.83 ± 0.33^c^	1666.19 ± 0.22^b^	−92.77 ± 0.38^b^


*Note:* Means with different superscripts in the same column are significantly different (Tukey′s test, *p* ≤ 0.05). Two‐way ANOVA indicated significant main effects of extraction method and substitution level, as well as their interaction (*p* ≤ 0.05).

Abbreviations: EAE, enzymatic‐assisted extraction; UAE, ultrasound‐assisted extraction; UAEE, ultrasound‐assisted enzymatic extraction.

The back extrusion test mimics the deformation of food as it occurs in the mouth or under stress and strain conditions during processing [42] and can also be related to the organoleptic and viscoelastic characteristics of the product [6]. In back extrusion analysis, firmness, as described in Figure 2, represents the maximum force needed to cause a deformation in the mayonnaise structure mimicking what occurs between the tongue and palate during oral processing, and reflects the thickness or “body” of the product [43]. Adhesiveness represents the amount of energy needed to overcome the attractive force between the mayonnaise and the surface material, such as the detachment from a spoon or probe, and is quantified as the negative work during probe withdrawal [44]. Consistency indicates the strength of internal bonds within the mayonnaise structure and can be correlated to how the mayonnaise spreads under applied force.

In this study, the firmness of mayonnaise was significantly affected by both extraction method and substitution level, with a strong interaction effect (*p* < 0.0001). Firmness generally increased in all formulations containing 5%–50% egg yolk substitution, except for samples with 50% UAE, which did not show an increment. Compared with the control (i.e., 63.50 N), EAE formulations exhibited firmness increment ranging from 56% to 95.3%, whereas UAEE formulations showed even greater increases, ranging from 37% to 163.4% (Table 2). These findings demonstrate that substitution with lyophilized MO seed protein extracts can enhance mayonnaise texture, particularly when proteins are obtained via EAE or UAEE methods, highlighting the importance of optimizing both extraction technique and substitution level.

**Figure 3:**
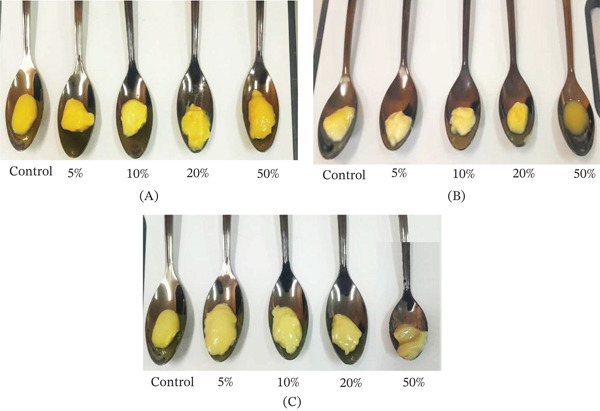
Mayonnaise formulations with different substitution levels of egg yolk. (A) Substitution with proteins from enzymatic‐assisted extraction (EAE), (B) substitution with proteins from ultrasound‐assisted extraction (UAE), and (C) substitution with proteins from ultrasound‐assisted enzymatic extraction (UAEE).

The consistency of the control sample (741.23 N·s) was lower than that of EAE and UAEE formulations, which exhibited significantly higher consistency across all substitution levels (Table 2 and Figure 3). Consistency is a textural characteristic that is reported to be positively correlated with smoothness, creaminess, and desirable mouthfeel [45]. The higher firmness and consistency observed with EAE and UAEE formulations may be attributed to the functional properties of the protein extracts, including water‐holding capacity and oil‐holding capacity. Previous studies have shown that such functional attributes can contribute to improved emulsion stability by enhancing protein adsorption at the oil–water interface and facilitating electrostatic interactions that reduce interfacial tension [46], thereby maintaining the firmness and consistency of the mayonnaise. Although these mechanisms were not directly measured in the present study, they provide a plausible explanation for the functional outcomes observed. A good balance between WHC and OHC is indicative of the amphiphilic surface characteristics and emulsion stability capacity [46, 47]. Amphiphilic particles, as explained by Böker et al. [48], possess distinctive hydrophilic and hydrophobic surface regions, which enable them to readily adsorb at the oil–water interface. This adsorptive property reduces the interfacial tension by a Pickering‐type mechanism to prevent coalescence [6].

Furthermore, we hypothesize that these characteristics may promote balanced wettability at the oil or water phase and contribute to a densely packed interfacial film that improves gel‐like consistency and firmness [49]. Similar observations were reported by Armaforte et al. [50] for mayonnaise produced from chickpea protein isolate, where increased firmness, adhesiveness, and consistency were attributed to the protein emulsion capacity and its role in structuring the continuous phase.

In the UAE formulation, 10%–20% protein inclusion enhanced firmness, consistency, and adhesiveness compared with the control. However, this trend reversed at 50% substitution, where all three textural attributes were significantly lower than the control (Table 2 and Figure3B). This implies that the threshold for incorporating UAE proteins into mayonnaise formulation was at 20%, and oversubstitution of egg yolk negatively affected the texture parameters of the mayonnaise. The reduced textural quality at higher substitution levels is likely due to the low water‐holding capacity of UAE protein extracts, induced by cavitation and cell disruption during UAE (Section 2.3.1), whereby more hydrophobic regions are exposed and reduce the protein–water interaction. According to Bianchi and Simonato, [51] and Dickinson [52], proteins with low water‐holding capacity are less effective at binding water within the continuous phase of the emulsion. This can lead to a less viscous and more fluid product, thereby reducing the overall consistency, thickness, and potentially affecting adhesion. This was also observed for 50% UAE mayonnaise (Figure 3B and Table 2).

**Figure 4:**
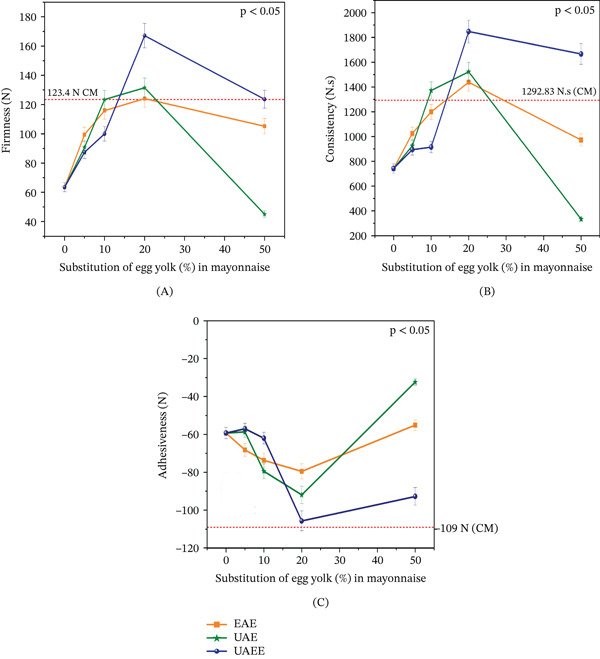
Effect of egg yolk substitution (%) on the textural properties of mayonnaise formulated with protein extracts from sprouted *Moringa oleifera* seeds. (A) Firmness, (B) consistency, and (C) adhesiveness. Ultrasound‐assisted extraction (UAE), enzymatic‐assisted extraction (EAE), and ultrasound‐assisted enzymatic extraction (UAEE). The commercial mayonnaise (CM) is included as a benchmark reference. Two‐way ANOVA revealed significant main effects of extraction method and substitution level, as well as their interaction (*p* < 0.05). Error bars represent standard deviation (*n* = 3).

Typically, CM is formulated using a variety of bulking and emulsifying agents, including thickeners, gelling agents, and viscosity modifiers, which help achieve the product texture that consumers prefer [53]. In this study, CM exhibited a firmness value of 123.4 N, consistency of 1292.83 N·s, and adhesiveness of −109.00 N (Figure 4). When compared across the substitution level, formulations with 20% replacement of egg yolk with EAE, UAE, and UAEE proteins matched or exceeded the standard set by CM for firmness and consistency. Implying that, at 20% substitution, all the protein extracts can be used to produce mayonnaise with consumer‐accepted firmness and consistency attributes. With regards to adhesion, a high absolute value indicates a stickier product [54]. In this regard, all the mayonnaise formations were less adhesive than CM, suggesting that the product was less sticky and separated more easily from the contact surface. Among the protein extracts, 20% replacement of egg yolk with UAEE proteins achieved adhesiveness comparable with CM, indicating its potential to replicate texture in commercially accepted full‐fat mayonnaise. These findings highlight the feasibility of partial egg yolk replacement with sprouted MO seed protein extracts, particularly those obtained via UAEE, which were associated with desirable textural properties in mayonnaise.

### 3.2. Emulsion Droplet Size of Mayonnaise

Emulsion droplet size analysis was conducted to assess the influence of protein source and the level of egg yolk substitution. Droplet size is indicative of the efficiency of protein adsorption at the oil–water interface, which occurs in three sequential stages: (i) transport of protein molecules to the interface, (ii) unfolding via partial denaturation to expose hydrophobic core residues, and (iii) formation of a cohesive viscoelastic film that protects droplets against coalescence [46]. The LD measurements of water‐dispersed emulsions assessed droplet size distribution, with smaller, more uniform values indicating improved emulsion stability [55]. In this study, the extraction method and level of egg yolk substitution influenced droplet size distribution (Table 3).

**Table 3:** Droplet size distribution in formulated mayonnaise.

Extraction method	Substitution level (%)	Droplet Size Distribution			Span
		D_10_ (*μ*m)	D_50_ (*μ*m)	D_90_ (*μ*m)	
UAE	5	54.74±0.03^j^	121.24±0.01^l^	201.36±0.05^l^	1.21±0.00^j^
	10	126.84±0.01^d^	251.27±0.03^g^	388.35±0.12^h^	1.04±0.00^l^
	20	105.83±0.02^e^	390.24±0.14^a^	896.20±0.10^a^	2.03±0.00^c^
	50	79.27±0.02^g^	269.35±0.05^f^	587.65±0.01^d^	1.89±0.00^d^

EAE	5	52.78±0.21^k^	154.23±0.02^j^	306.07±0.12^j^	1.64±0.00^e^
	10	49.95±0.07^l^	141.30±0.20^k^	276.00±0.02^k^	1.60±0.00^f^
	20	99.76±0.21^f^	222.90±0.27^i^	372.27±0.03^i^	1.22±0.00^i^
	50	136.76±0.09^c^	285.41±0.08^d^	456.82±0.17^g^	1.12±0.00^k^

UAEE	5	141.76±0.21^b^	318.37±0.12^c^	534.20±0.19^f^	1.23±0.00^h^
	10	145.80±0.19^a^	333.86±0.21^b^	566.22±0.01^e^	1.26±0.00^g^
	20	58.21±0.06^i^	243.77±0.14^h^	607.03±0.01^c^	2.25±0.00^b^
	50	62.33±0.08^h^	279.18±0.01^e^	725.37±0.03^b^	2.37±0.00^a^


*Note:* UAE: protein extracts from ultrasound‐assisted extraction; EAE: protein extracts from enzymatic‐assisted extraction; UAEE: protein extracts from ultrasound‐assisted enzymatic extraction. Based on each formulation section, means with different superscripts in the same column are significantly different (*p* value ≤ 0.05).

**Figure 5:**
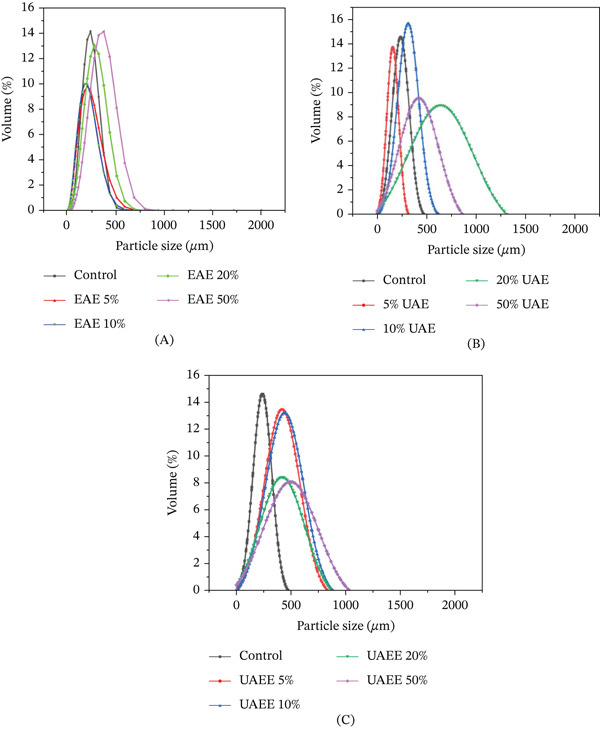
Particle size distribution of mayonnaise. (A) Mayonnaise made with proteins from enzyme‐assisted extraction (EAE), (B) mayonnaise made with proteins from ultrasound‐assisted extraction (UAE), and (C) mayonnaise made with proteins from ultrasound‐assisted enzymatic extraction (UAEE).

All formulations exhibited a monomodal particle distribution (Figure 5), with percentile descriptors (*D*_10_, *D*_50_, and *D*_90_) summarizing the spread. However, the distributions were asymmetrical and skewed toward larger particle sizes, indicating the presence of coarse droplets that may compromise emulsion stability. In high‐oil emulsions such as mayonnaise, monomodal distributions are only desirable when centered around smaller droplet sizes, as this reduces heterogeneity and limits the coarse fraction responsible for coalescence and creaming [56]. Among the percentile descriptors, *D*_90_ is particularly important because it captures the coarse fraction of droplets that governs coalescence and creaming behavior ([57]). Thus, reductions in *D*_90_ provide the strongest evidence of improved emulsion stability in this study. At low substitution levels, EAE and UAE formulations showed significant reductions in *D*_90_ compared with the control (298.19 *μ*m), indicating enhanced stability. Specifically, 5% UAE and 10% EAE substitutions yielded smaller *D*_90_ values than the control mayonnaise (298.19 *μ*m), reflecting efficient droplet disruption during homogenization and rapid adsorption of protein extracts at the oil–water interface. Such reductions in droplet size are likely associated with faster interfacial migration, enhanced protein unfolding, and stronger interfacial film formation, ultimately contributing to increased viscosity, opacity, and emulsion stability [58]. However, droplet size reduction and distribution uniformity (span) did not always coincide (Table 3). For example, at low substitution levels, UAE at 5% and EAE at 10% produced smaller mean diameters but broader spans, indicating that fine droplets coexisted with coarse fractions. In contrast, UAE at 10% showed both reduced mean diameter and a narrower span, reflecting a more uniform droplet population.

**Figure 6:**
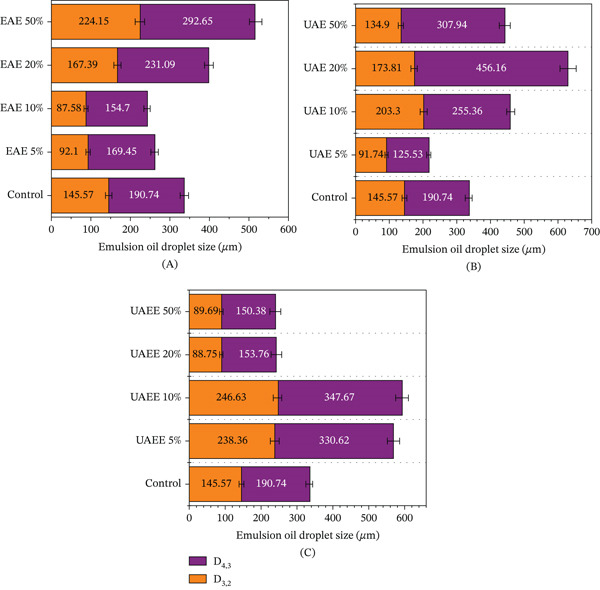
*D*_4,3_ and *D*_3,2_ emulsion droplet size distribution of mayonnaise. (A) Mayonnaise made with proteins from enzyme‐assisted extraction (EAE), (B) mayonnaise made with proteins from ultrasound‐assisted extraction (UAE), and (C) mayonnaise made with proteins from ultrasound‐assisted enzymatic extraction (UAEE).

At higher substitution levels, *D*_90_ increased, reflecting larger droplets and reduced stability. Notably, UAE and UAEE formulations consistently exhibited higher *D*_90_ values, suggesting slower protein migration to the interface for emulsion stabilization. This trend was further supported by droplet size distribution breadth (span), particularly UAEE at 20% and 50% substitution (span > 2.25), which exhibited broader distributions, consistent with the presence of coarse droplets and reduced stability. By comparison, EAE at 50% produced smaller mean diameters but a span statistically indistinguishable from the control, indicating that fine droplet formation did not reduce the coexistence of coarse fractions. This observation aligns with previous reports that mean diameter alone is insufficient to infer emulsion stability, which depends on distribution breadth and coarse droplet behavior [37]. The significant effects of the extraction method and substitution level on both *D*_90_ and span were confirmed by two‐way ANOVA (*p* < 0.05). Other factors, such as protein purity, solubility, continuous‐phase viscosity, and oil distribution, may also influence droplet growth and stability (D. [59]). Despite these confounding variables, the functional role of UAEE in emulsion formation was evident from the weighted mean diameter (*D*_3,2_) and volume‐weighted mean diameter (*D*_4,3_) values, which reflected measurable interfacial activity (Figure 6).

#### 3.2.1. Effect of Protein Inclusion on Surface and Volume Weighted Mean Diameter

Figure 6 illustrates the weighted mean diameter (*D*_3,2_) and volume weighted mean diameter (*D*_4,3_) of the oil droplets in the formulated mayonnaise. *D*_3,2_ represents the average surface area of a droplet exposed to the continuous phase per unit weight of the emulsion, providing an estimate of the total interfacial area that contributes to a smoother adhesive texture [60]. Therefore, *D*_3,2_ is highly sensitive to smaller droplet sizes. In contrast, *D*_4,3_ is more sensitive to the presence of larger droplets and therefore serves as a strong indicator of destabilization phenomena such as flocculation, coalescence, and creaming in concentrated emulsions like mayonnaise [61]. Together, these two distributions provide insight into emulsion characteristics such as texture and susceptibility to phase separation [62].

Mayonnaise with 5%–10% substitution with EAE protein extracts had significantly (*p* value ≤ 0.05) smaller *D*_3,2_ distribution than the control (Figure 6A). This observation can be attributed to the enzymatic treatment, which can reduce protein molecular size and increase conformational flexibility due to the presence of random coils in the protein structure, enabling faster migration and denser packing at the oil–water interface, even at low substitution levels ([63]). This enhanced adsorption decreased total interfacial area and improved droplet coverage, effectively encapsulating the oil phase and limiting coalescence. As a result, the emulsion contained finer, more uniformly distributed droplets that formed a closely packed network [63]. However, as the level of egg yolk substitution increased from 20% to 50%, a converse increase in oil droplet surface area was observed.

This may be attributed to antagonistic interactions between EAE proteins and residual egg yolk components, which disrupted interfacial stability and promoted aggregation of initially smaller droplets into larger ones. Similar trends were reported by Choi et al. [8] in mayonnaise formulated with pea protein–xanthan gum conjugates, where higher substitution levels led to increased droplet aggregation, altering both emulsion microstructure and viscosity. Likewise, Mohammadi et al. [64] observed that higher inclusion of amaranth protein reduced emulsifying efficiency, resulting in polydisperse emulsions with larger, nonuniform oil droplets.

UAE proteins also had smaller *D*_3,2_ and *D*_4,3_ values at 5% egg yolk substitution, which represents a 36% reduction in the oil droplet size relative to the control (Figure 6B). The authors hypothesize that this reduction may reflect complementary effects between UAE proteins and egg yolk components, potentially arising from the greater total availability of surface‐active components (egg yolk phospholipids plus UAE proteins) or changes in continuous‐phase viscosity [65]. Ultrasonication induces the exposure of sulfhydryl groups and hydrophobic regions, thus enhancing the protein′s ability to migrate to the oil–water interface to reduce interfacial tension between the continuous and dispersed phases [66]. At low substitution levels, the hydrophobic characteristics of UAE proteins may complement the emulsifying action of egg yolk components, particularly phospholipids and hydrophobic proteins like lipovitellin and livetin, which form a dense, elastic interfacial network [2, 67]. However, at higher egg yolk substitution (i.e., 10%–50% UAE), the converse was observed. This may be due to the system becoming dominated by hydrophobic interactions, disrupting the critical balance between hydrophilic and hydrophobic domains required for optimal emulsion stability ([68]). This imbalance can lead to interfacial crowding and protein–protein aggregation, ultimately compromising emulsion stability and promoting droplet coalescence ([63]).

A converse observation was seen for UAEE mayonnaise formulation at 5% and 10%, where *D*_3,2_ was significantly (*p* value ≤ 0.05) larger than the control (Figure 6C). Nevertheless, this pattern changed at 20% and 50% substitution, where values were 38% smaller than the control. This reduction in droplet size at higher egg yolk substitution suggests that UAEE extracts at higher inclusion levels can enhance the protein migration to the oil–water interface, promote molecular unfolding, and enable denser adsorption of protein molecules around droplets. Collectively, these occurrences effectively lowered interfacial tension and enhanced emulsification. At these higher substitution levels, the increased availability of protein at the oil–water interface and dense interfacial coverage can be attributed to a phenomenon known as “interfacial jamming,” where tightly packed protein particles restrict oil droplet movement to prevent droplet coalescence [52, 69]. Furthermore, the increase in UAEE inclusion in the mayonnaise can increase total solids and viscosity of the continuous phase and likely contribute to the stabilization of smaller droplets. Collectively, these occurrences can enhance energy dissipation during homogenization, limit droplet collision, and enable rapid interfacial adsorption of protein [70].

Currently, an increase in the level of replacement of egg yolk with plant protein extracts has been reported to increase the *D*_4,3_ value, indicating a shift toward larger volume‐weighted droplet size [64]. An effect associated with emulsion destabilization and coalescence [36]. In this study, the data (Figure 6) suggest a threshold effect for EAE, UAE, and UAEE inclusion in mayonnaise formulation, beyond which the mayonnaise is more susceptible to droplet coalescence and gravitational separation during storage [34]. Therefore, among the formulations, 5%–10% EAE, 5% UAE, and 20%–50% UAEE achieved low *D*_3,2_ alongside a moderate increase in *D*_4,3_, a combination indicative of finer droplet distribution, slower rate of coalescence, and therefore, implies an improved microstructural integrity and possibly long‐term stability when compared with the control. However, these correlations do not establish direct causality, as other factors such as protein concentration, continuous‐phase viscosity, and oil distribution may also influence the observed improvements. Future studies should control for these variables to confirm the specific contribution of droplet size.

### 3.3. Emulsion Stability of Mayonnaise

**Figure 7:**
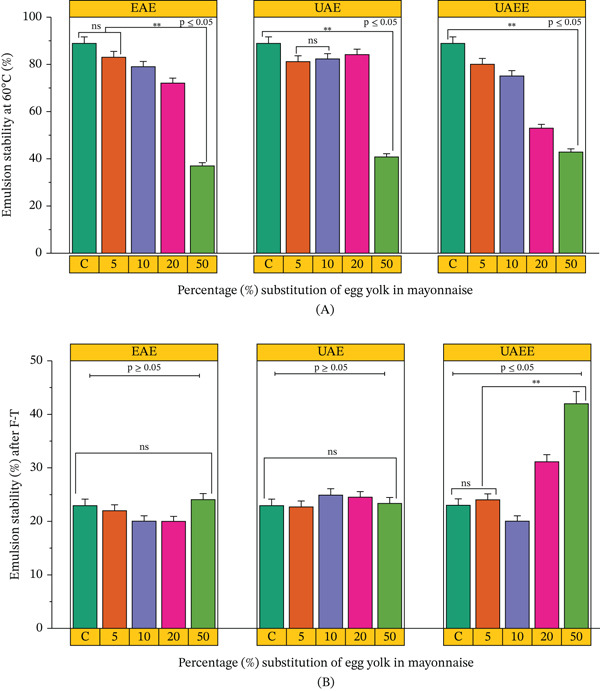
Emulsion stability of the formulated mayonnaise under different conditions. (A) Emulsion stability at 60°C, (B) emulsion stability after freezing and thawing (F‐T). ns: not significantly different (*p* value > 0.05), ^**^: significant difference (*p* value ≤ 0.05), C: control (mayonnaise made with only egg yolk as protein), UAE: ultrasound‐assisted extraction, EAE: enzymatic‐assisted extraction; UAEE: ultrasound‐assisted enzymatic extraction.

Stability is a vital quality index of mayonnaise as it determines both consumer acceptability and shelf stability. To assess the formulation′s resilience to emulsion destabilization, this study evaluated emulsion stability under two temperature conditions, specifically, thermal emulsion stability (60°C) and F‐T cycles (freezing at −20°C and thawing at 25°C) (Figure 7).

#### 3.3.1. Thermal Emulsion Stability of Mayonnaise

An increase in temperature can accelerate the movement and collision frequency of oil droplets, making them more prone to coalesce ([68]). This coalescence leads to the separation of the dispersed and continuous phases, a phenomenon known as emulsion breakdown, which is visually unappealing and indicates a spoiled product. Under heat exposure, the control formulation with only egg yolk exhibited the highest stability (89%), retaining its structure with minimal phase separation even after centrifugation (Figure 7A). This can be attributed to the unique proteins in egg yolk, such as phospholipids (lecithin) and lipoproteins, which are effective in reducing oil–water phase separation [10]. Additionally, lecithin retains its functional stability at temperatures up to 60°C–65°C, particularly when sugar and salt are present [1]. In contrast, thermal emulsion stability was reduced when there was a partial replacement of egg yolk between 5% and 50% with the UAE, EAE, and UAEE protein extracts (Figure 7A). However, this was less pronounced in UAE formulations at moderate substitution levels (20%) compared with EAE and UAEE formulations within similar ranges.

UAE proteins exhibited a slightly higher thermal emulsion stability than UAEE and EAE proteins (Figure 7A). The thermal stability observed in UAE formulations may reflect differences in how UAE influences protein functionality. A trend consistent with reports that applying ultrasound to extract proteins can enhance the emulsifying properties of MO seed protein [71]. However, emulsion stability under heat stress can be influenced by multiple factors, such as protein solubility, aggregation state, and continuous‐phase viscosity [4, 52]. Nonetheless, the emulsion stability of UAE only partially offsets the loss of egg lecithin in the formulation because at 50% substitution, the absence of yolk phospholipids outweighed the initial stability benefits of UAE protein, resulting in stability values of 40.8% (UAE), which was even lower than UAEE at 50% substitution (i.e., 43%). This indicates that UAE proteins contribute positively to thermal stability at intermediate levels but cannot fully compensate for the functional role of egg yolk phospholipids at higher substitution levels.

The reduced stability in EAE and UAEE may be associated with protein structural changes arising from partial hydrolysis, which can potentially increase protein structure flexibility but reduce the cohesive strength of the interfacial film under heat stress [49]. Although this interpretation is consistent with the literature, it remains tentative in the present study, as detailed structural characterization of EAE and UAEE is presented separately. The lowest value for thermal stability of the formulations was obtained at 50% substitution of egg yolk with UAE (54.1%), EAE (58.42%), and UAEE (51.6%). These findings imply that even though the protein extraction method may induce thermal stability in the proteins, its significance in maintaining emulsion under heat stress diminishes, especially at higher egg yolk substitution. The findings in this study are similar to those in previous studies by Mohammed et al. [10] for whey proteins, Jeong and Oh. [72] for aquafaba, and Rahmati et al. [73] for soy milk, where plant‐based protein mayonnaise, without sufficient phospholipids, yielded emulsions more susceptible to droplet coalescence under thermal stress.

#### 3.3.2. F‐T Stability of Mayonnaise

**Figure 8:**
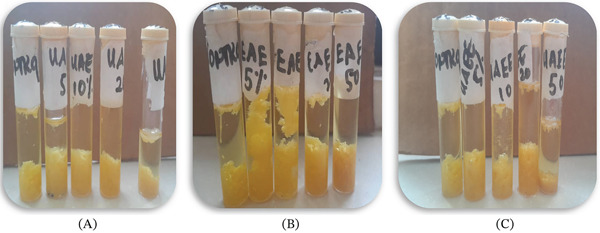
Images of mayonnaise after freezing at −20°C and thawing at 25°C for 30 min. (A) Mayonnaise made with proteins from ultrasound‐assisted extraction (UAE), (B) mayonnaise made with proteins from enzyme‐assisted extraction (EAE), and (C) mayonnaise made with proteins from ultrasound‐assisted enzymatic extraction (UAEE). Starting from the left, the control is followed by 5%, 10%, 20%, and 50% substitution.

F‐T cycles represent destabilization through the crystallization of water in the continuous phase, whereby ice crystal formation and expansion can disrupt the structural integrity of encapsulated oil droplets [74]. This disruption is seen during the thawing cycle as syneresis, whereby the compact gel structure is replaced by a watery, curdled (Figure 8) and visually unappealing matrix with layers of liquid or oil [74, 75].

Results from F‐T stability revealed that mayonnaise formulations incorporating UAE, UAEE, and EAE‐derived proteins as partial egg yolk replacements exhibited emulsion breakdown similar to the control. All samples, including the control, did not contain any stabilizer and thus underwent similar destabilization events under similar freezing and thawing conditions. As shown in Figure 7B, statistical analysis revealed no significant differences (*p* ≥ 0.05) between EAE and UAE formulations and the control across substitution levels. UAEE formulations at 50% substitution exhibited significantly greater F‐T stability than the control (*p* ≤ 0.05), with an 82.6% increase calculated based on reduced oil separation after thawing. The improved F‐T stability observed in UAEE formulations may reflect the ability of the protein extracts to immobilize water within the emulsion matrix, thereby limiting ice crystal growth and reducing oil separation during thawing. This interpretation aligns with protein‐stabilized interfacial films reported in high internal phase emulsions (HIPEs), where strong interfacial networks entrapped water and oil droplets and effectively prevented coalescence during F‐T cycles [11]. However, this observed resilience was time‐dependent. Relative to the other formulations that destabilized after 5 min of thawing, UAEE′s complete destabilization was observed after 30 min. This indicates that the initial stability advantage may diminish over extended thawing.

### 3.4. Color Attributes of Formulated Mayonnaise

All measured color parameters (L*, a*, b*, and YI) differed significantly across treatments (*p* value ≤ 0.05) (Table 4). The L* value, which quantifies lightness on a scale from 0 (*black*) to 100 (*white*), declined progressively with increasing egg yolk substitution, indicating a darker visual appearance in reduced‐egg formulations. Specifically, 20%–50% UAE substitution resulted in a 5.1%–13.15% reduction in L* relative to the control, which was 65.82 ± 0.45, whereas EAE formulations within the same substitution range showed a 3.84%–9.51% decrease. UAEE treatments exhibited the most pronounced decline at similar levels of substitution and ranged from 9.58%–19.88%. This reduction in L* reflects diminished light scattering and surface reflectance, both of which influence consumer perception of freshness and quality.

**Table 4:** Color attributes and pH of mayonnaise.

Extraction method	Substitution level (%)	Color parameters			YI	pH
		L*	a*	b*		
UAE	5	66.87 ± 0.01^ab^	−8.35 ± 0.01^a^	30.56 ± 0.01^cd^	65.29 ± 0.0^f^	5.16 ± 0.01^d^
	10	67.65 ± 0.01^a^	−8.37 ± 0.01^a^	27.71 ± 0.01^g^	58.52 ± 0.0^h^	5.22 ± 0.03^d^
	20	62.45 ± 0.05^d^	−7.92 ± 0.01^bc^	31.14 ± 0.15^b^	70.90 ± 0.3^d^	5.32 ± 0.00^c^
	50	57.16 ± 1.10^f^	−6.36 ± 0.15^f^	30.12 ± 0.28^cd^	78.40 ± 0.3^a^	5.38 ± 0.00^bc^

EAE	5	65.78 ± 0.01^bc^	−8.45 ± 0.01^a^	33.95 ± 0.21^a^	74.00 ± 0.4^b^	5.16 ± 0.0^d^
	10	65.36 ± 0.01^c^	−8.03 ± 0.01^b^	28.90 ± 0.18^ef^	63.62 ± 0.4^g^	5.19 ± 0.00^d^
	20	63.29 ± 0.01^d^	−7.62 ± 0.00^d^	29.57 ± 0.03^de^	66.69 ± 0.0^e^	5.21 ± 0.00^d^
	50	59.56 ± 0.25^e^	−6.66 ± 0.03^e^	30.56 ± 0.00^cd^	73.11 ± 0.3^bc^	5.16 ± 0.00^d^

UAEE	5	66.25 ± 0.02^bc^	−7.79 ± 0.23^cd^	30.21 ± 0.00^cd^	65.08 ± 0.1^f^	5.04 ± 0.06^e^
	10	66.13 ± 0.01^abc^	−8.29 ± 0.01^a^	28.87 ± 0.50^f^	63.25 ± 1.0^g^	5.07 ± 0.06^e^
	20	59.51 ± 0.15^e^	−5.91 ± 0.01^g^	25.54 ± 0.01^h^	72.24 ± 0.2^c^	5.43 ± 0.01^b^
	50	52.73 ± 0.67^f^	−4.70 ± 0.05^h^	27.16 ± 0.49^g^	73.71 ± 0.4^b^	5.57 ± 0.01^a^


*Note:* UAE: protein extracts from ultrasound‐assisted extraction; EAE: protein extracts from enzymatic‐assisted extraction; UAEE: protein extracts from ultrasound‐assisted enzymatic extraction. Based on each formulation, means with different superscripts in the same column are significantly different (*p* value ≤ 0.05).

Abbreviation: YI, yellowness index.

A similar pattern was observed for a* values, which generally declined across all formulations as substitution increased, irrespective of the protein extract type. This reduction in redness can be attributed to the loss of egg yolk carotenoids, notably lutein and zeaxanthin [76] which imparts the warm hue characteristic of traditional mayonnaise. Plant‐derived protein extracts typically lack these pigments or contain alternative chromophores with lower red intensity, leading to a perceptible shift toward paler or more neutral tones. Similar findings have been reported in studies using chickpea, broad bean, and lupin protein isolates, where increased substitution levels led to noticeable changes in lightness and lower redness due to the natural color of the plant proteins and their interaction with oil and acidulants during emulsification [77].

The YI, derived from L* and b*, reflects the formulation′s combined brightness and yellow hue intensity. The control sample recorded the highest YI (174.00 ± 1.19), consistent with its full complement of yolk‐derived carotenoids efficiently dispersed within the mayonnaise matrix. The largest reduction in YI occurred at 10% substitution for UAE, EAE, and UAEE (66%, 63%, and 63%, respectively), lower than the control, revealing a pigment threshold where the diminished concentration of yoke chromophores significantly affected the yellowness of the mayonnaise. Although extraction techniques such as sonication and EAE are known to enhance the release of phenolic compounds, including anthocyanins, their chromatic properties are primarily absorbed in the blue–violet spectrum [78, 79]. Therefore, possible pigmentation derived from UAE, EAE, and UAEE protein extracts have a limited contribution to yellowness.

### 3.5. Effect of Protein Extracts on pH of Mayonnaise Formulations

The pH of the mayonnaise remained within a relatively narrow range across substitution levels since all formulations contain the same amount of acidulant (Table 4). The slight but consistent increase in pH observed with higher levels of egg yolk substitution can be attributed to the diminished buffering capacity of the formulation. Egg yolk proteins, particularly phosvitin and livetin, are the primary contributors to buffering in the acidic pH range due to their abundance of ionizable groups, especially phosphate residues [67]. As their concentration decreases with increasing levels of UAE, EAE, and UAEE protein extracts, their ability to resist pH fluctuations also decreases. Although plant proteins contain ionizable side chains such as carboxyl, amino, and imidazole groups [46, 80], their buffering effect within the acidic pH range is relatively weak and does not compensate for the loss of egg yolk protein. Consequently, the formulation becomes more sensitive to acid–base shifts, increasing pH values from 5.04 to 5.57 (Table 4). The pH values observed were consistent with those reported by Bolarin and Oke, [16] for mayonnaise formulated with moringa seed flour as a partial egg yolk substitute.

This pH range classifies the formulated mayonnaise as a low‐acid food. Regulatory agencies, including the US FDA (21 CFR Part 114) and Codex Alimentarius (CAC/RCP 23‐1979) define low‐acid foods as having a pH > 4.6 [81, 82]. Typically, mayonnaise has a pH ranging between 3.60 and 4.00 [75]. In the present study, all substituted formulations had a pH of 5.04–5.57, indicating that acid alone could no longer serve as the primary microbial control hurdle. At such pH values, acid‐tolerant spoilage organisms (e.g., yeasts, molds, and lactic acid bacteria) may proliferate more rapidly, and pathogenic microorganisms such as *Clostridium botulinum*, *Salmonella* spp., and *Listeria monocytogenes* may survive [83].

To ensure microbial safety during processing and storage, formulations containing UAE, EAE, and UAEE must include additional preservative components. These may include incorporating approved antimicrobial agents and/or utilizing modified atmosphere packaging (MAP).

## 4. Conclusion and Future Research

In this study, plant‐based mayonnaise analogues were produced by substituting 5%–50% of egg yolk with proteins extracted from sprouted MO seeds. The extraction method influenced the proteins′ functional performance, particularly their interfacial behavior, with direct impact on the mayonnaise texture, emulsion droplet size, color, pH, and emulsion stability. Incorporating 20% EAE, UAE, or UAEE protein extracts significantly improved firmness, consistency, and adhesiveness compared with the control. At this optimal level, UAEE mayonnaise matched a commercial benchmark in terms of firmness and consistency and adhesiveness. Moreover, 5%–10% EAE, 5% UAE, and 20% UAEE formulations exhibited smaller *D*_4,3_ and *D*_3,2_ values than the control, indicating finer droplet size distributions. Color analysis showed that the decline in YI with increasing egg yolk substitution was primarily due to the reduced proportion of egg yolk in the formulation, the main source of carotenoids responsible for the characteristic yellow color. The pH increased slightly but consistently with higher substitution, and formulation pH ranged between 5.04 and 5.57. Mayonnaise with 5%–10% egg yolk substitution using UAE, EAE, or UAEE proteins showed moderate stability under both thermal and F‐T conditions.

This study provides insights into how sprouted MO seed proteins extracted through UAE, EAE, and UAEE can successfully replace up to 20% of egg yolk in mayonnaise while meeting or exceeding the textural performance of commercial products, reducing droplet size, and partially maintaining color. These results contribute to the knowledge of sustainable, functional plant protein utilization into high‐oil emulsions and provide insights into reduced‐egg mayonnaise formulated from MO seed proteins. Future work should focus on optimizing the formulations for shelf stability, validating consumer acceptance, and integrating safe, effective antimicrobial strategies tailored to low‐acid emulsions.

## Funding

This study was supported by the University of Ghana Nestlé Scholarship for Research Excellence through Nestlé SA.

## Conflicts of Interest

The authors declare no conflicts of interest.

## Data Availability

The dataset supporting the conclusions of this article is included in this article.
